# 

**Published:** 1848-04

**Authors:** 


### THE WESTERN JOURNAL OF MEDICINE AND SURGERY.

### EDITORS.

DANIEL DRAKE, M. D.,

PROFESSOR OF PATHOLOGY AND THE PRACTICE OF MEDICINE IN THE UNIVERSITY OF LOUISVILLE.

LUNSFORD P. YANDELL, M. D.,

PROFESSOR OF CHEMISTRY AND PHARMACY IN THE SAME

THOMAS W. COLESCOTT, M. D

